# Multipoint monitoring of amplitude, frequency, and phase of vibrations using concatenated modal interferometers

**DOI:** 10.1038/s41598-022-07354-6

**Published:** 2022-03-08

**Authors:** Kalipada Chatterjee, Venugopal Arumuru, Dhananjay Patil, Rajan Jha

**Affiliations:** 1 Nanophotonics and Plasmonics Laboratory, School of Basic Sciences, Indian Institute of Technology Bhubaneswar, Khurda, 752050 India; 2 Applied Fluids Group, School of Mechanical Sciences, Indian Institute of Technology Bhubaneswar, Khurda, 752050 India

**Keywords:** Aerospace engineering, Civil engineering, Mechanical engineering, Optical sensors

## Abstract

Concatenated modal interferometers-based multipoint monitoring system for detection of amplitude, frequency, and phase of mechanical vibrations is proposed and demonstrated. The sensor probes are fabricated using identical photonic crystal fiber (PCF) sections and integrated along a single fiber channel to act as a compact and efficient sensing system. Each identical probe acts as a modal interferometer to generate a stable interference spectrum over the source spectrum. In the presence of an external dynamic field about each probe, the probes respond independently, producing a resultant signal superposition of each interferometer response signal. By analyzing the resultant signals using computational techniques, the vibration parameters applied to each interferometer are realized. The sensing system has an operation range of 1 Hz-1 kHz with a sensitivity of 51.5 pm/V. Such a sensing system would find wide applications at industrial, infrastructural, and medical fronts for monitoring various dynamic physical phenomena.

## Introduction

Real-time monitoring of mechanical vibrations over multiple locations enables structural health monitoring^1^, posture recognition^2^, dynamic field sensing^3,4^, and industrial surveillance. Such mechanical vibrations are characterized by physical parameters, such as frequency, amplitude, and relative phase, which differ over a broad range depending on the measurement field's functionality. Conventional electronic vibration sensors have prominently achieved detection of the aforementioned vibration parameters at many fronts. However, the performance of these devices is influenced by power fluctuations, environmental temperature variations, and stray electromagnetic field interference. Alternatively, optical fiber-based vibration sensors have proven to be a potential class of devices that function on the principle of modulation of intensity, wavelength composition, or phase of the propagating light in the presence of an external measurand field. These sensors are compact, accurate, reconfigurable, corrosion-resistant, temperature tolerant, and immune to electromagnetic fields^5,6^.

All-fiber interferometric techniques like Sagnac interferometer^7,8^ Mach–Zehnder interferometer^9^, Michelson interferometer^10^, and their combinations^11^ have been reported for distributed contactless sensing of vibrations driven by dynamic displacements and strains. However, these sensors are bulky, require precise alignments, and require multiple sensing channels. Besides, vibration detection has been demonstrated by measuring the external vibration-induced back-scattered light in conventional fiber using optical time-domain reflectometry (OTDR) techniques that rely on the modulation of the phase (Φ-OTDR)^12,13^ polarization state (POTDR)^14^, and beam frequency (BOTDR)^15^ of the propagating wave in the presence of external perturbation. However, these sensing systems incur diminished sensitivity over long-range distributed sensing and require additional expensive components like pulsed lasers, electro-optic modulators (EOM), and erbium-doped fiber amplifiers (EDFA). Also, FBG based vibration interrogators have been utilized to develop distributed vibration sensors^16,17^. However, FBG based distributed sensing systems require additional semiconductor amplifiers and additional test mass that make them bulky. Besides, the identical FBG probes need to be separated by large distances to distinguish their resonant frequencies and are intolerant towards external temperature fluctuations^18^. Dynamic measurement of instantaneous frequency, amplitude, and phase of the vibration at multiple locations using a single fiber channel has remained challenging and elusive. Simultaneous instantaneous phase monitoring at multiple locations can enlighten the complex structural vibrations associated with unsteady aerodynamic loads and can also be used to infer instantaneous peak vibrations, which are otherwise difficult to be monitored using conventional Fourier transform-based averaging techniques^19^.

This paper proposes and demonstrates a multipoint vibration sensing technique for detecting instantaneous frequency, amplitude, and phase of vibrations about each interferometer by implementing solid-core photonic crystal fiber (SCPCF) based modal interferometry principle. Such interferometry principle involves fabricating fiber configurations with specialty waveguides such as SCPCF, enabling excitation and recombination of waveguide modes resulting in stable interference spectra over the source spectrum. Identical fiber configurations concatenated along with a fiber channel act as independent interferometers to detect the vibrations at each point. The SCPCF has a better guiding mechanism and inherent significant temperature tolerance, which makes it superior to conventional fiber^20,21^. The resultant signal is analyzed using simple computational techniques to determine the information of vibrations about each interferometer. A similar system has been previously investigated but for static mechanical strain and not for real-time dynamic sensing^22^. The proposed system enables real-time monitoring of dynamic strain about multiple points.

## Results

### Sensor structure and working principle

**Figure 1:**
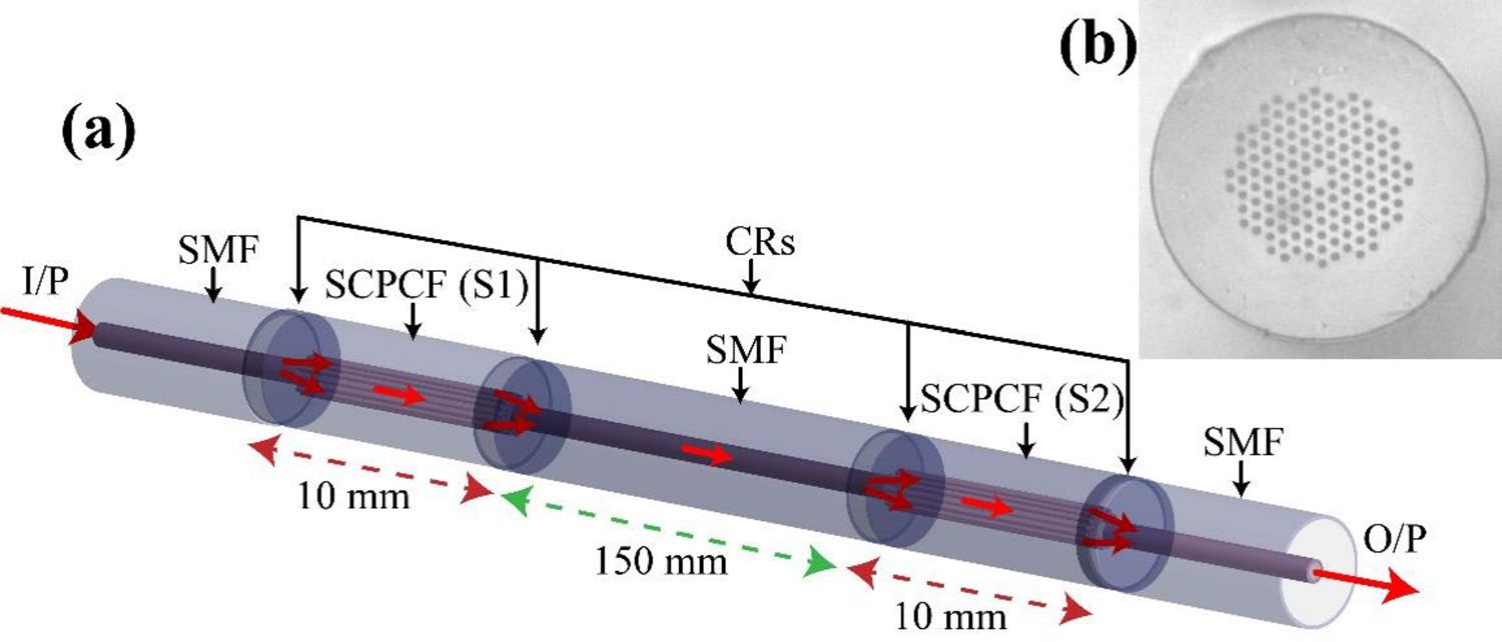
Schematic representation of integrated sensor probes for vibration sensing. Each PCF section acts a modal interferometer to enable sensing. Here, I/P: Input light, SMF: Single mode fiber, SCPCF: Solid core photonic crystal fiber (S1 and S2), CR: Collapse region, O/P: Output or transmitted light. (b) SEM image of the SCPCF used for fabrication of the interferometers.

In our approach, identical sections of commercially available solid-core photonic crystal fiber (SCPCF) are concatenated along a single-mode fiber (SMF) length (Fig. 1a) to act as the sensing system. The SCPCF used as sensor probe has an outer diameter of 125 μm with a core diameter of 8 μm, surrounded by a sixfold symmetric arrangement of voids with a diameter of 3.1 μm and pitch of 6.6 μm (LMA-8, by NKT Photonics) as shown in Fig. 1b. Equal lengths of SCPCF sections are concatenated along a single fiber channel at a distance of 150 mm. The concatenation or splicing process of SCPCF with SMF collapses the air holes of SCPCF near the splicing joint such that the core-cladding boundary becomes indistinct. This region is called the collapse region.

**Figure 2:**
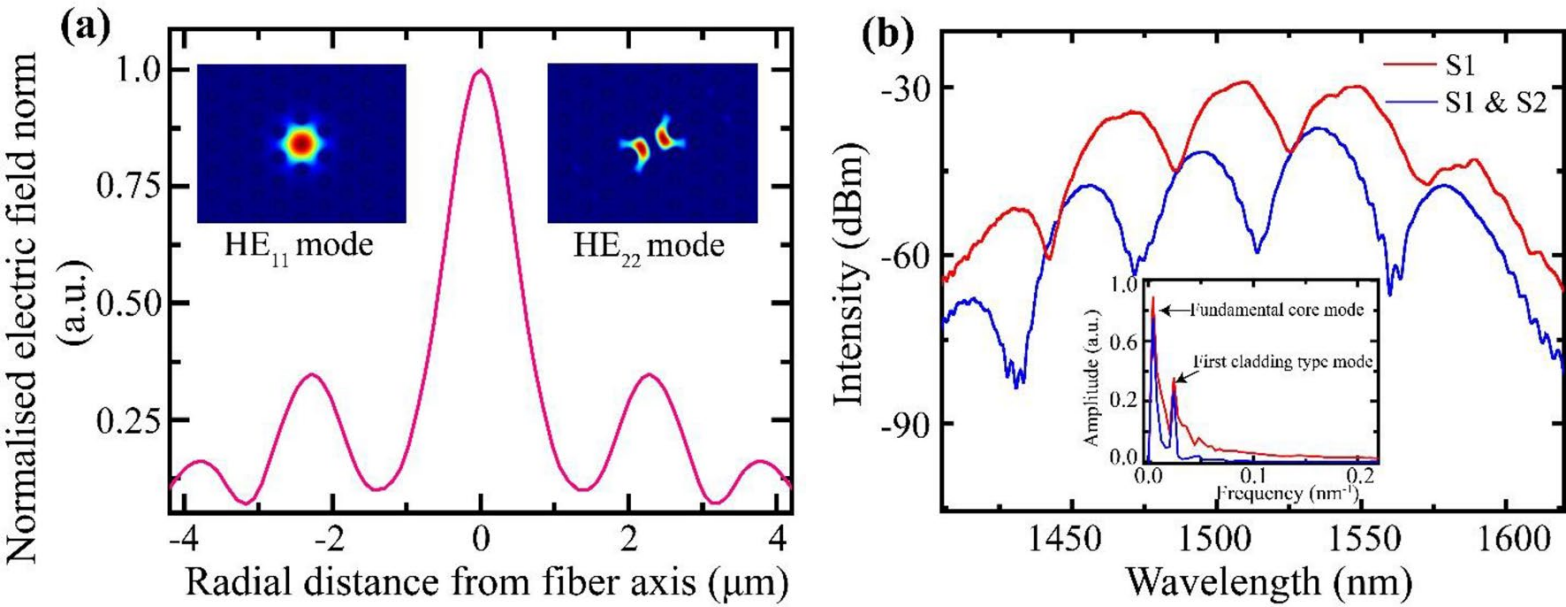
(**a**) Simulated electric field distribution about the SCPCF core (inset) Mode profile of HE_11_ core mode and HE_22_ cladding type mode. (**b**) Characteristic transmission spectra of the array of sensors for only first sensor (S1) (red curve) and both sensors along the SMF (blue curve).

When light from a broadband source is coupled to the sensing system, at the collapse region, the light diffracts, broadens, and excites higher-order guided modes along with the fundamental core mode of the SCPCF (S1) fiber (as represented in Fig. 1). Here, the fabricated probes have a collapse region length (l) of ~ 212 μm. The resultant spot size of the propagating mode through the collapse region at the SCPCF core is calculated using the formula^23^

1 $$ w = w_{0} \sqrt {1 + \left( {\lambda l/\pi n_{c} w_{0}^{2} } \right)^{2} } $$

where w and w_o_ (5 μm) are beam waist radius at the PCF and SMF core, respectively, λ is the wavelength of light (1550 nm), n_c_ is the refractive index of pure silica (1.444). The spot size (2w) is calculated to be ~ 31 μm. Due to the large mode field area under propagation over the collapse region and inherent modal characteristics of the SCPCF, the modes with azimuthal symmetry are supported^24^. The resultant mode field distribution about the SCPCF core simulated using COMSOL Multiphysics is shown in Fig. 2a. Thus, from the calculated mode field area at the SCPCF core, it is concluded that the HE_11_ core mode and the quasi-HE_22_ cladding-like modes are excited^23^ (inset Fig. 2a). The effective refractive indices of the core and cladding modes are 1.4354 and 1.4352, respectively. These modes propagate with an effective wave vector (k) that depends on the wavelength and their effective refractive indices along the SCPCF section. The guided modes diffract, overlap, and superpose at the second collapse region while getting coupled to the SMF core. A similar mechanism takes place at the second SCPCF (S2) as both the interferometers are identical. The resultant intensity variation over the source spectrum at each modal interferometer, I_j_(λ), can be written as^25^,

2 $$ I_{j} \left( \lambda \right) = I_{cr} + I_{cl} + 2\sqrt {I_{cr} I_{cl} } \cos \left( {\phi \left( \lambda \right)} \right) $$

where I_cr_ and I_cl_ are spectral intensity distributions of core and cladding modes of PCF, φ is the phase difference between the two modes given by^26^,

3 $$ \phi \left( \lambda \right) = \frac{{2\pi L\left( {n_{cr}^{eff} - n_{cl}^{eff} } \right)}}{\lambda } = \frac{2\pi \Delta nL}{\lambda } $$

where L is the length of PCF, λ is the free space wavelength of light, $n_{cr}^{eff}$ and $n_{cl}^{eff}$ are the effective refractive indices of core and cladding modes of PCF, respectively and Δn is the difference between the effective modal refractive indices. The resultant characteristic spectra of the sensing system for a single interferometer and for two interferometers in series are shown in Fig. 2b. The maxima of the spectra occur for the condition φ = 2Nπ or ΔnL = Nλ that is dependent on the wavelength of light and length of the interferometers at a constant value of Δn and N, which is an integer. As the interferometers are spliced under identical conditions, the spectrum of concatenated interferometers is similar to that of a single interferometer except with weak overriding peaks in the former that arises from the negligible intermodulation products in a typical sensor array. The negligible loss in the average power is attributed to the splice loss at the collapse region. The power loss can be controlled by altering the length of the collapse region to prevent the excitation of lossy modes. Besides, it can be compensated using a source with higher output power, unlike OTDR techniques requiring additional fiber amplifiers. Fast Fourier transform (FFT) of the characteristic spectra reveal the two excited SCPCF modes, that is, the fundamental core mode and the first order cladding-type mode, that overlap along its length (inset Fig. 2b) to generate the characteristic spectra.

In the presence of an external static strain field about the interferometer, the length of the interferometer changes due to field-induced bending, and the magnitude of change in length depends on the amplitude and direction of the applied strain. Due to the change in interferometer length, the wavelength values corresponding to the interference maxima condition readjust. This causes a shift in the maxima peak positions that results in modulation of I_j_(λ) over the spectral range. In the presence of alternating strain or vibrational field of specific amplitude and frequency about an interferometer, the bending of the interferometer becomes periodic. As a result, each interference maximum shift periodically over a range of wavelength (Δλ) values about the initial maximum position or wavelength (λ_o_), resulting in dynamic modulation of I_j_(λ). The corresponding sensitivity (S_j_(λ_o_)), that is the net shift (Δλ) of maximum wavelength about λ_o_ of intensity I_j_(λ) with respect to measurand field, for each interferometer (j) is represented as,

4 $$ S_{j} \left( {\lambda_{o} } \right) = \frac{{\partial I_{j} \left( \lambda \right)}}{{\partial X_{j} }} $$

where X is the external vibration field that can be given as X = X_o_ sin (ωt + θ) with X_o_, ω, and θ being the amplitude, frequency, and initial phase of vibration, respectively. Experimentally, a single maximum position (λ_o_) is tracked, and its peak shift (Δλ) is recorded in the presence of the external field. Under the simultaneous operation of multiple concatenated interferometers in transmission mode, the resultant sensitivity (S_n_(λ_o_)) about the tracked maximum position is a summation of the individual sensitivities and can be represented as,

5 $$ S_{n} \left( {\lambda_{o} } \right) = \mathop \sum \limits_{j} \frac{{\partial I_{j} \left( \lambda \right)}}{{\partial X_{j} }} $$

where j = 1, 2, … where j = 1 corresponds to the sensor nearest to the source end. However, due to the extra path length traversed by the signal of S1, as the path lengths are equal up to the detector beyond S2, the signal at S1 incurs a definite phase lag. Theoretically, the value of phase delay acquired by a light wave of wavelength 1550 nm along the SMF length of 150 mm is calculated to be ~ 0.4π radians. As a result, the system's response is unique for different dynamic strain field parameters at each interferometer. For example, the composite signal for applying vibration of frequency f1 about S1 and f2 about S2 is different from that of the signal in which vibrations of frequency f2 is applied about S1 and f1 about S2. Consequently, the transmitted signal is a linear superposition of the individual interferometer responses, and its nature depends on the phase lag in the signals of sensors acquired by virtue of their position along the fiber channel. The resultant real-time sensitivity is influenced by the amplitude, frequency, and relative phase of vibrations about each interferometer. In such cases, wherein multiple interferometers are concatenated to act as independent sensors, the separation between the interferometers can be varied as per requirement^27^, and the phase lag between the individual responses can be controlled. Using suitable computational techniques, the vibration parameters at each interferometer can be realized to enable localization of the external field.

### Multipoint monitoring

**Figure 3:**
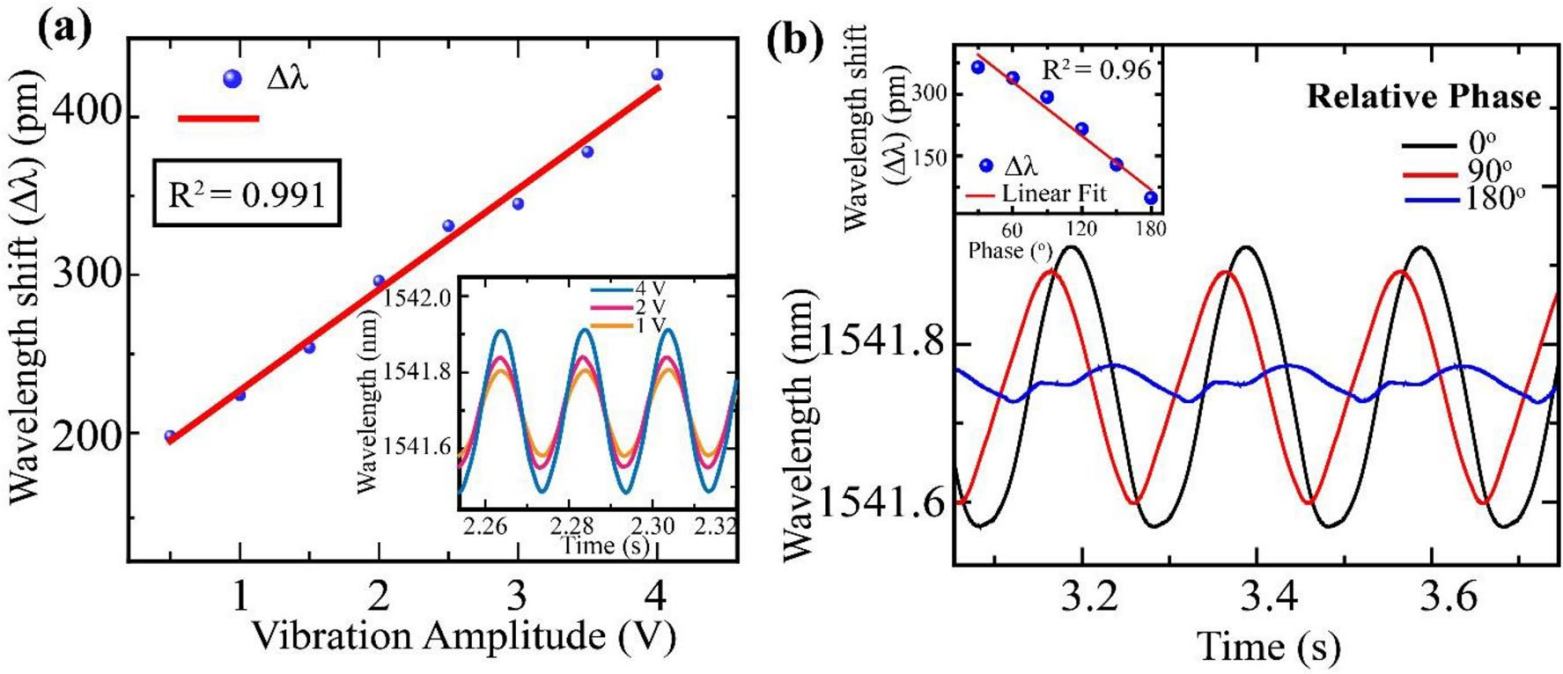
(**a**) Characteristic plot for wavelength shift of resultant signal versus vibration amplitude at 50 Hz. (inset) Real time resultant signal for varying only the amplitude of vibrations applied about S2 while keeping all other parameters identical about S1 and S2 at 50 Hz. (**b**) Real time resultant signal for applying vibrations of 10 Hz and fixed amplitude (1.5 V) but with initial relative phase difference about S1 and S2. The relative phase difference between the vibrations is provided in the figure. (inset) Characteristic plot for wavelength shift of resultant signal versus relative phase between vibration components.

Figure 3 shows the results of the characterization of the sensing system. Firstly, in-phase vibrations of 50 Hz are applied about both interferometers, and only the amplitude of vibrations at S2 is varied (see Fig. 3a), keeping the amplitude fixed at S1. As the amplitude of vibrations is increased, the maxima of the interference spectrum shift over a broader range of wavelengths (inset of Fig. 3a). From the calibrated curve, it is observed that the sensitivity or magnitude of peak shift increases linearly (R^2^ value = 0.991) with the rise in applied vibrational amplitude. This enables the detection of minute changes in vibrational amplitude over a broad range. The rate of change in sensitivity with applied vibration voltage is found to be 51.5 pm/V, that is significant to determine the variation in the strength of vibration. Similar observations are made when vibrations of varying amplitudes are applied about S1, keeping the vibrations’ amplitude fixed about S2. Thus, by recording the amplitude of the resultant signal, the strength of the vibrations about the interferometers can be determined.

Further, we investigate the response of the proposed sensing system for applying vibrations of constant frequency and constant amplitude about S1 and S2 but with different relative phase differences in vibrations about the sensors. It is carried out by varying the initial phase of vibrations at S1. It is observed that the resultant real-time sensitivity decreases with an increase in the relative phase difference between the vibrations about S1 and S2 as shown in Fig. 3b. The rate of change in sensitivity with the relative phase difference in vibrations about the sensors is evaluated to be − 21.8 pm/ 10-degree phase difference between vibrations. The variation in sensitivity has significant linearity (R^2^ = 0.96) . The sensitivity is maximum when the relative initial phase is 0° and minimum for that of 180°. The cause is attributed to the constructive and destructive superposition of signals at 0° and 180° relative phase between the vibrations, respectively. By determining the resultant peak shift (Δλ), the phase relation between the vibrations at the sensors is realized.

**Figure 4:**
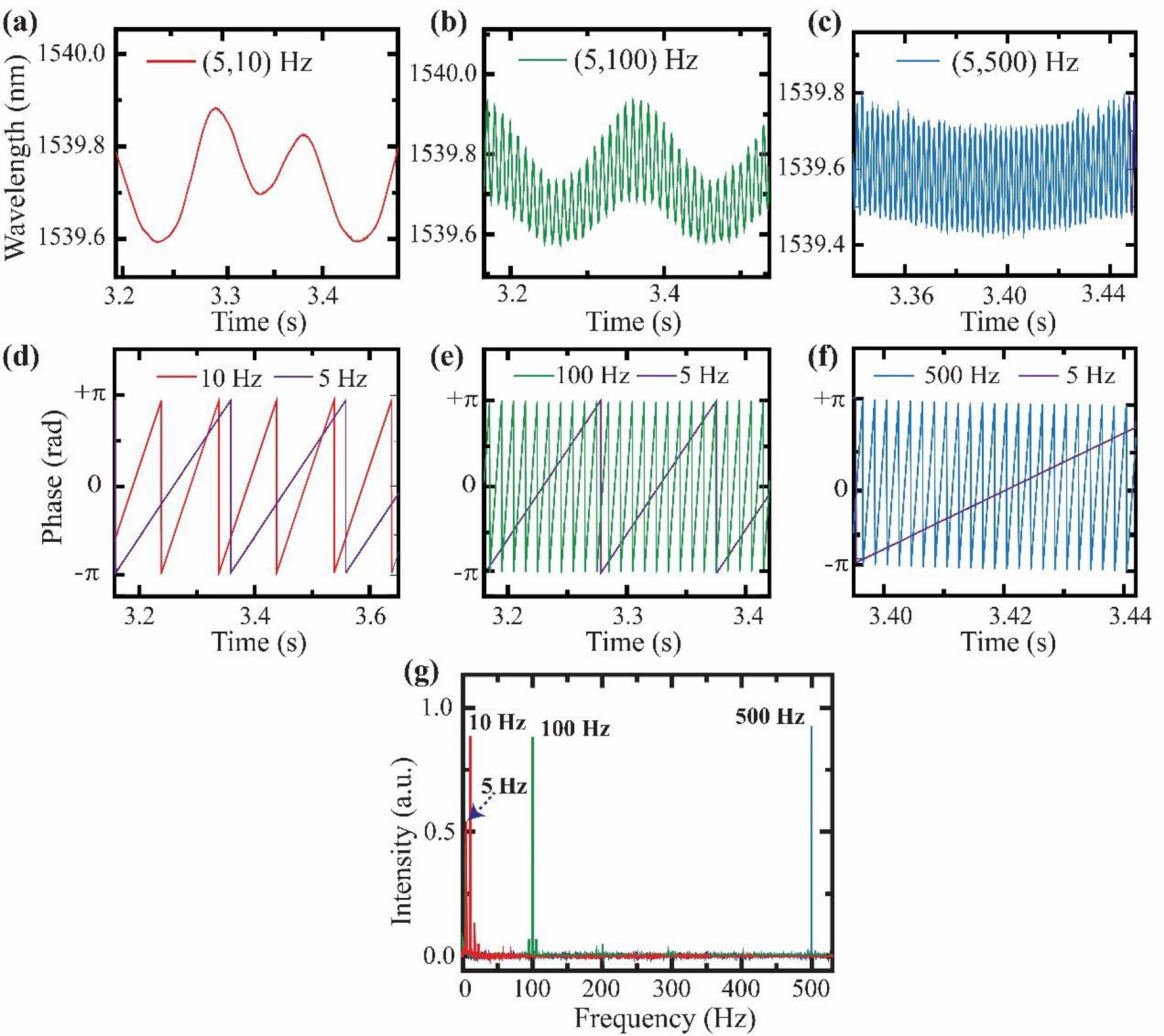
(**a**)–(**c**) Real time resultant signal for varying only the frequency of vibrations applied about S2 while applying vibrations of 5 Hz about S1 and keeping all other parameters identical about S1 and S2. (**d**)–(**f**) Instantaneous phase of individual signals about S1 and S2 for the signals shown in (**a**)–(**c**). (**g**) FFT of the signals represented in (**a**)–(**c**) that show sharp peaks at the applied vibration frequencies.

Subsequently, the values of amplitude and initial phase of vibrations about both interferometers are fixed at 1.5 V and 0°, respectively. The frequency of vibrations at S1 is fixed at 5 Hz while the vibration frequency is varied about S2 (see, Fig. 4a–c). The frequency of the shift in maxima wavelength or sensitivity about the initial value corresponds to the frequency of the applied vibration. It is observed from the time-series signal that the sensitivity at S1 is modulated by the sensitivity at S2 and the resultant periodic shift in the interference spectra is a superposition of the individual signals, as predicted by theoretical analysis. However, there is a definite phase relationship between the two superposing signals due to their position along the fiber length. To analyze the signal and determine the vibration parameters about the sensors, computational techniques associated with signal processing are used. The complex signal is decomposed into individual components using Wavelet transform (WT) signal processing tool. Using continuous WT methods, individual frequencies are extracted, and their instantaneous phase is evaluated (see Fig. 4d–f). The fast Fourier transform (FFT) of the complex signals are computed and depicted in Fig. 4g. The FFT shows sharp peaks at each frequency with a high signal to noise ratio (SNR). Similar observations are made when the frequency of vibrations is kept fixed about S2 and is varied at S1. The frequency range over which the proposed sensing system is tested is 1 Hz-1 kHz, wherein it showed favorable results. Technically, the sensitivity of the system with respect to frequency is influenced by the resonance frequency of the system. About the resonant frequency, the sensitivity of the system is maximum and tends to behave non-linearly. In this case, the system's resonant frequency depends on sensor probes positioning with respect to the transducers that affect the tension along the waveguide channel^28^. The sensing arrangement can be altered to reconfigure the resonant frequency conditions. Hence, the system can be configured to respond to frequencies beyond 1 kHz as the sensing probes are optical waveguides that are not influenced by any electrical noises. Thus, the sensing system enables monitoring the instantaneous frequency, amplitude, and phase of each vibration independently about multiple points.

### Computational analysis

**Figure 5:**
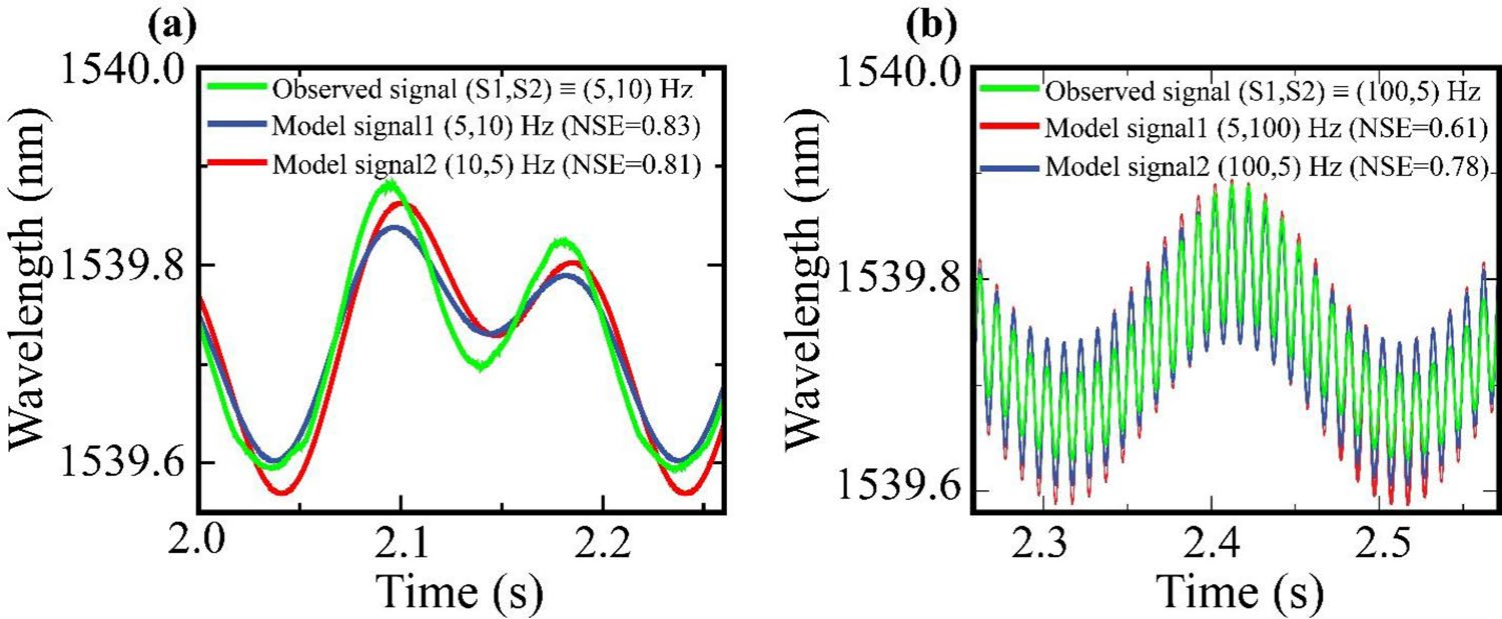
(**a**) Comparison of real time observed signal of operating S1 at 5 Hz and S2 at 10 Hz with corresponding analytical model signals. (**b**) Comparison of real time observed signal of operating S1 at 100 Hz and S2 at 5 Hz with corresponding analytical model signals.

To achieve distributed or multipoint sensing with the proposed system, it is essential to identify the location and parameters of vibrations about each interferometer when both interferometers are subjected to different vibrations. Certainly, the frequency components in the signal and their corresponding strength are determined from WT and FFT techniques. In order to determine the position of these frequency components about S1 and S2, a computational technique based on signal comparison is used. As discussed, the signal of operating S1 at frequency f1 and S2 at frequency f2 and the signal obtained by reversing the frequencies at S1 (at f2) and S2 (at f1) are different due to the inherent phase lag in the light wave while propagating from S1 to S2. Based on this fact, model analytical signals of varied combinations are simulated by considering similar phase lag between the signal components as in the proposed system. The recorded real-time signals are then compared with the simulated signals and their corresponding Nash–Sutcliffe efficiency (NSE) value is evaluated. The NSE coefficient is used for such comparison because it is a widely implemented and accepted coefficient for data analysis in environmental studies^29–31^. The NSE value in our technique is calculated for the observed signal with respect to each model signal using the equation,

6 $$ NSE = 1 - \frac{{\mathop \sum \nolimits_{T} \left( {S_{n}^{m} - S_{n}^{o} } \right)^{2} }}{{\mathop \sum \nolimits_{T} \left( {S_{n}^{o} - \overline{{S_{n}^{o} }} } \right)^{2} }} $$

where $S_{n}^{m}$ is the model signal, $S_{n}^{o}$ is the observed signal and $\overline{{S_{n}^{o} }}$ is the mean of $S_{n}^{o}$ over time. The model signal, which on comparison with the observed signal yields the highest NSE value, provides information about the signal components at each interferometer. As the interferometers are identical, this technique provides a simple way to determine the frequency of vibration about each interferometer. In Fig. 5, two such analyses are shown. For instance, Fig. 5a depicts the comparison of the observed signal for operating S1 at 5 Hz and S2 at 10 Hz with corresponding model signals. From the calculated NSE values, it is evident that the observed signal correlates more with the equivalent model signal instead of the other model signal in which the frequencies’ order is reversed. In the case of concatenating more than two interferometers along the SMF length, the model analytical signals need to be formulated more precisely. This can be achieved by characterizing the sensing system for more signal combinations to obtain sufficient datasets as the training set.

**Figure 6:**
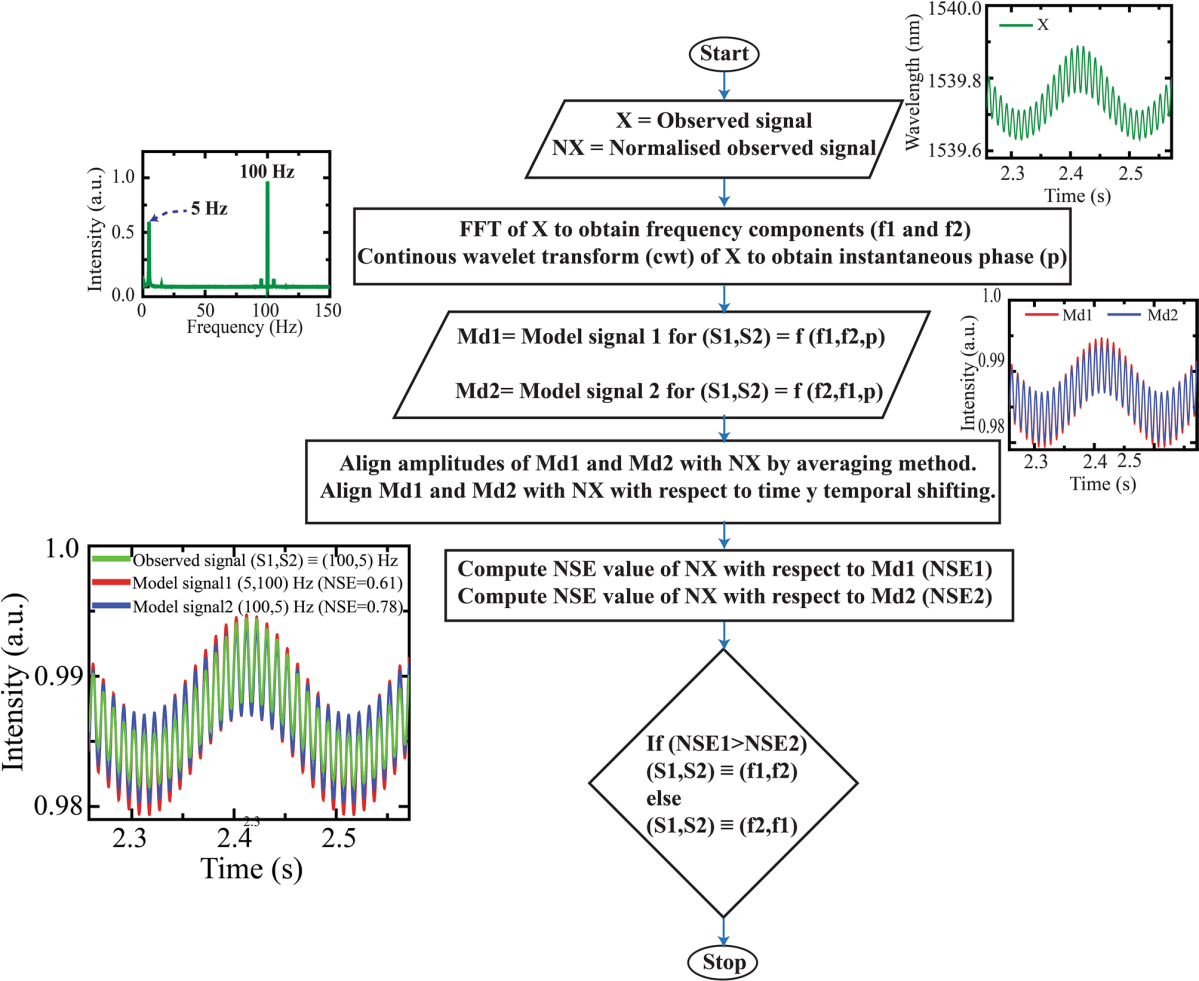
Flowchart representation of the computational technique for signal analysis.

The flowchart of the computational program for signal analysis is depicted in Fig. 6. In this method, firstly, the frequency components in the recorded composite signal (X) are determined using FFT and their instantaneous phases are obtained by taking WT of the signal. These frequency values (f1 and f2) and instantaneous phase (p) information are used to construct the model signals (Md1 and Md2). The model signals are designed as sinusoidal signals with a definite phase relationship between the components to become comparable with the composite signals. The model signals are expressed in all possible sequences of superposition of the frequency components. In this case, as there are two frequency components, so the signals are possible for two combinations of the components that is (f1,f2) and (f2,f1). Subsequently, the amplitude of the model signals are made comparable with that of the observed signal by comparing their average values and then made them to align with respect to the time axis. The NSE values are calculated for X with respect to Md1 and Md2 and compared for determining the sequence of frequency components along the fiber channel about the interferometers. The developed computational technique is tested for multiple experimental datasets, and it predicts the sequence of frequency components quite accurately. Besides, the technique can be extended for more than two sensors by simply modifying the model signals depending on the sensing system’s response. Further, the technique analyzes the model signals with the normalized observed signal, which makes the analysis independent of vibration amplitude.

## Discussion

In this work, we report modal interferometry-based multipoint dynamic field sensing system for various applications. Previously, such a technique has been used for static field monitoring at different locations. However, in this work, identical interferometers (similar interference period) are used to reduce the cross talks between the interferometers that influence the SNR values in sensing utilities. In case of dynamic field monitoring, SNR needs to be high. In this work, due to the independent operation of identical interferometers, dynamic fields about each interferometer can be monitored with significant efficiency. The proposed system can be extended for more than two sensors. As long as the interferometers are identical, the maximum number of PCF interferometric sensors that can be concatenated in a single channel depends on the power of the optical source and on the splice losses of the sensor probes along the fiber channel. In order to track a peak, sufficient power should be contained within it to be detected by the detector. To give a number, if a broadband light source of power 10 dBm and a detector with sensitivity down to -80 dBm are used, then under plausible conditions, about 6 identical interferometers can be concatenated along the fiber channel as the splice loss is ~ 7 dBm along with other insertion losses. This number can be improved with a higher power source or by optimizing the length of the collapse region.

## Conclusion

In conclusion, we proposed and demonstrated a compact multipoint vibration sensing technique enabled by concatenated modal interferometers for simultaneous real-time monitoring of instantaneous frequency, amplitude, and phase of vibration along a single fiber channel. The vibration parameters about each interferometer can be determined by computational technique for the localization of the source of vibrations and facilitating simultaneously monitoring multiple units. The interferometers are identical and operate independently to generate a resultant output possessing information of the perturbing field at different locations. Besides, the interferometers' size can be reconfigured while keeping them identical, and the distance between their positions can be altered as per requirement. The proposed system can be tuned as per requirement and can be easily extended for more than two interferometers in series. Monitoring multiple optical sensors concatenated along a single fiber channel using a single interrogation unit is economical and can be extended to develop a sensors network. The modal interferometry technique for distributed sensing offers a platform for designing robust and sensitive single-channel vibration sensing over long distances as an alternative to the existing expensive commercial techniques, for industrial applications and structural surveillance. On a specific note, this sensing system can be used to monitor fans and compressors at different locations in heating, ventilation, and air conditioning (HVAC) industries and automotive industries to monitor running motors used in the manufacturing processes. Besides, such a system can be used to survey fluid flow in factories by installing them along different pipelines^32^.

## Methods

### Numerical Simulations

2D light propagation through the SMF-PCF-SMF fiber channel is simulated by finite element method using wave optics module in COMSOL Multiphysics. The parameters used for FEM simulations are scaled accurately concerning the experimental setup. The mesh element size was set to be smaller than 1/12 of the area of the fiber channel. The computation is carried to obtain two modes for the spectral range. The mode profiles of SCPCF (shown in Fig. 2a inset) are simulated for using the SCPCF features similar to the one used for experimental purposes.

### Sensor fabrication

For preparing the first SCPCF-SMF section (S1), at first, SCPCF of length 10 ± 0.01 mm is taken whose ends are prepared by thoroughly cleaning and cleaving perpendicularly using precision fiber cleaver (Sumitomo, FC-6RS). The cleaved end of the SCPCF is then spliced with SMF at optimized parameters using a fusion splicer (Fujikura, 80S). The length of the interferometers is kept as 10 mm so that the period of the interference pattern is broad and the peaks are sufficiently distinct to avoid overlapping. Similarly, another SCPCF section (S2) of equal length (10 ± 0.01 mm) is spliced along the length of the same SMF at a distance of 150 mm along the SMF length. Alternatively, if two separate interferometers are fabricated individually and concatenated along the fiber length, then the fiber length between the interferometers will be fragile and incur splice losses. The distance between the interferometers is kept as 150 mm such that each interferometer operates independently and can be clamped thoroughly on both sides, to reduce the effect of mechanical noises.

### Experimental charcatreization of sensing system

To characterize the sensor probes, light from a broadband Super-luminescent Light Emitting Diode (SLED) source (Thor Labs S5FC1005S) emitting about a central wavelength of 1552 nm is launched through the in-line interferometers. The resultant characteristic interference spectrum of the sensing is recorded using a spectrum analyzer (OSA, by Yokogawa). The spectrum (Fig. 2b) is first recorded for a single interferometer (S1) (red line) and then for the two interferometers integrated in series (blue line).

**Figure 7:**
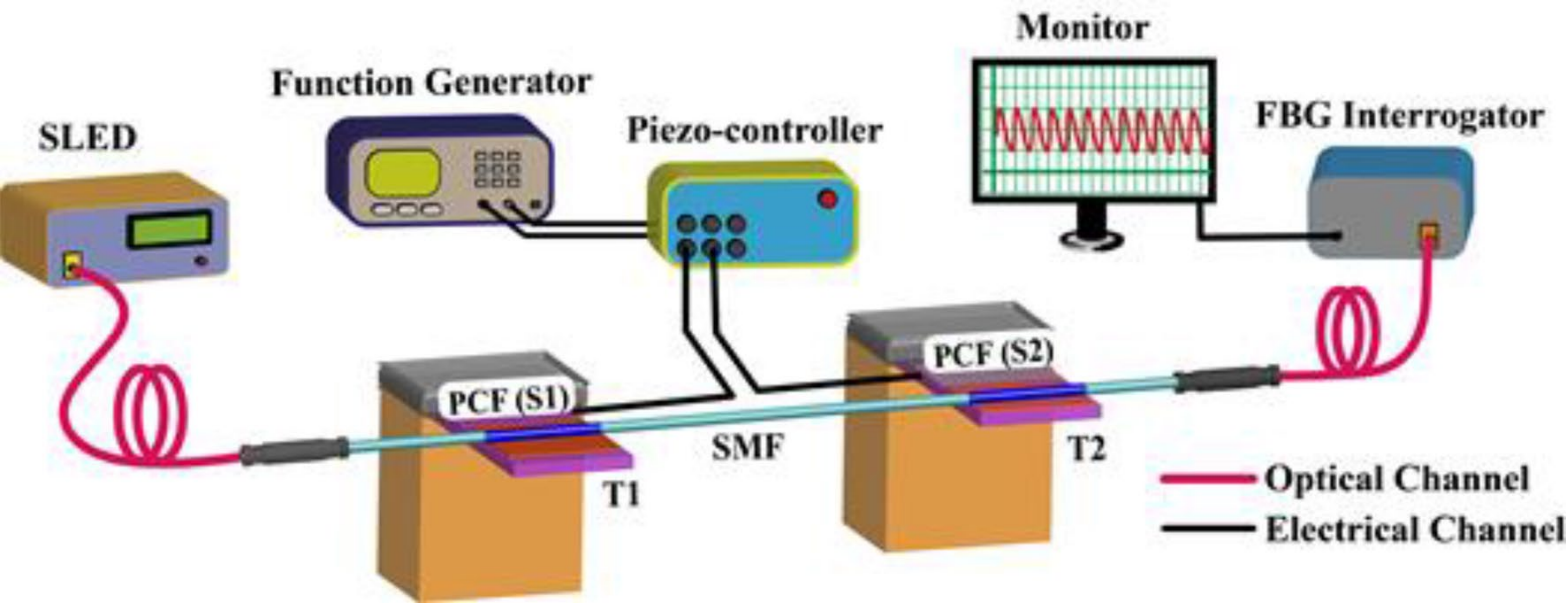
Schematic of the experimental setup for characterization of the proposed optical vibration sensing system.

For vibration monitoring, the interferometers (S1 and S2) are mounted on piezoelectric transducers (PZT: T1 and T2, respectively) to interrogate the interferometers with respect to external vibrations over real-time, as shown in Fig. 7. The PZTs are powered by a multi-channel piezoelectric controller (by Piezosystems). The piezo controller is driven by a function generator (by Tektronix) to control the vibration parameters of the PZTs to generate vibrations of varied frequencies and amplitudes. The sensor probes are kept straight to avoid strain or bend-induced losses along the interferometers' fiber length. For detecting the real-time signal, the transmitted spectra are fed to an FBG based high-speed wavelength interrogator (Ibsen, I-MON 256 HS), and the output is recorded on a computer via an ethernet interface. The applied mechanical vibrations bend the interferometers periodically depending on the frequency and amplitude of the vibrations. The periodic shift in maxima wavelength of transmission spectra, in the presence of external vibrations, is tracked for recording the real-time response of sensor probes. The parameters of vibrations that is amplitude, frequency, and initial phase, are varied about each interferometer, and the signals are recorded for analysis.
